# Prognostic value of PCT in septic emergency patients

**DOI:** 10.1186/s13613-016-0146-4

**Published:** 2016-05-21

**Authors:** Nicolas Peschanski, Camille Chenevier-Gobeaux, Lynda Mzabi, Rémy Lucas, Siham Ouahabi, Vianney Aquilina, Valéry Brunel, Guillaume Lefevre, Patrick Ray

**Affiliations:** Department of Emergency Medicine, Centre Hospitalo-universitaire de Rouen, 1 rue de Germont, 76000 Rouen, France; Department of Automated Biological Diagnosis, Hôpitaux Universitaires Paris Centre (HUPC) – Hôpital Cochin, Assistance Publique des Hôpitaux de Paris (AP-HP), Paris, France; Department of Emergency Medicine, Hôpitaux Universitaires Est Parisien - Hôpital Tenon, Assistance Publique des Hôpitaux de Paris (AP-HP), 4 rue de la Chine, 75020 Paris, France; Department of Biochemistry and Hormonology, Hôpitaux Universitaires Est Parisien - Hôpital Tenon, Assistance Publique des Hôpitaux de Paris (AP-HP), 4 rue de la Chine, 75020 Paris, France; Department of Biochemistry, Clinical Biology Institut, Centre Hospitalo-universitaire de Rouen, 1 rue de Germont, 76000 Rouen, France; Sorbonne Universités UMPC Université Paris 06, DHU Fighting Aging and Stress (FAST), Paris, France

**Keywords:** Procalcitonin, Sepsis, Emergency department, Prognostic

## Abstract

**Background:**

An accurate assessment of septic patients at risk for poor clinical outcomes is challenging for clinicians in the emergency department (ED).

**Objectives:**

We aimed to evaluate the prognostic value of procalcitonin (PCT) in septic patients in the ED for predicting death.

**Results:**

In a retrospective study, 188 septic patients (median age 63 [IQR 51–80]) of two French university hospitals were included. Patients who deceased within 30 days (20 %, *n* = 37) presented higher PCT value at admission (median 34.0 µg/L [5.0–71.9]) in comparison with the survivals (median 6.4 µg/L [4.1–13.1], *p* = 0.0005). ROC curve analysis indicated a moderate AUC of 0.686 [95 % CI 0.613–0.752] and an optimal PCT threshold value at 32.5 [95 % CI 21.8–43.3] µg/L that was associated with a 51 % [34–67] sensitivity, a 96 % [90–98] specificity, a 73 % [52–88] positive predictive value, and a 89 % [83–93] negative predictive value for death. Only 26 patients (14 %) had PCT values above this threshold (19 in the deceased group vs 7 in survival group, *p* < 0.0001). By multivariate analysis, only three variables remained significantly predictive of the death: personal history of cardiovascular disease (OR 3.1 [1.0–9.4], *p* = 0.046), the presence of severe sepsis/septic shock in the ER (OR 4.4 [1.3–12.3], *p* = 0.013), and a PCT level >32.5 µg/L (OR 36.0 [10.0–128.4], *p* < 0.0001). Similar results were obtained when considering the combined outcome death and/or admission in ICU.

**Conclusion:**

Elevated value of PCT at admission has moderate accuracy to identify poor outcome in ED septic patients in daily practice.

## Background

Sepsis is a life-threatening condition that arises when the host’s response to an infection injures its own tissues and organs [1]. The accurate identification of sepsis is one of the main challenges in emergency medicine. Despite advances in antibiotic therapy and modern life support, fatality rate of patients with sepsis has remained high worldwide (>30 %) [1, 2].

Early identification of patients at high risk of dying from sepsis may help initiate rapid and appropriate therapeutic interventions. However, signs of organ dysfunction may not be obvious for the physician’s at the time of presentation [3]. Thus, an accurate assessment of patients at risk for poor clinical outcomes is challenging for clinicians in the emergency department (ED).

Procalcitonin (PCT), the pro-hormone of calcitonin, is synthesized by thyroid cells. During sepsis, many tissues and immune cells become able to secrete PCT. The enhanced specificity of PCT along with the publication of numerous clinical and interventional studies has contributed to the growing interest and implementation of PCT in the ED as a biomarker of the systemic host response to bacterial invasion [3, 4]. Several studies have demonstrated that PCT may confer prognostic information in PAC [5] and in sepsis [6], and some have suggested the same in an ED. Recently, PCT-based sepsis diagnosis was demonstrated to be more reliable and discriminating than clinical sepsis diagnosis [7]. Furthermore, lactate and PCT might be complementary biomarkers for the risk stratification of ED patients evaluation [3]. Thus, PCT might be a good candidate to accurately identify ED septic patients with poor outcome.

Therefore, we aimed to evaluate the prognostic value of PCT in septic patients in the ED.

## Patients and methods

It was a retrospective study, which took place in the EDs of two university hospitals (Tenon hospital in Paris and Rouen), from July 2011 to December 2011. Both were urban adult ED and teaching hospitals with, respectively, 45,000 and 82,000 annual new patient attendances. Because of the observational design of the study, the ethical committee (CPP Ile-de-France Paris VI, Paris, France) authorized a waiver of informed consent. In these two hospitals, PCT is widely used by physicians’ in charge, for patients with suspected or confirmed infections, especially in suspicion of lower respiratory tract infections. However, no real guideline is associated with the result of PCT.

### Studied population

Patients ≥18 years old were included if they presented with a confirmed diagnosis of infection, and had a PCT measurement blood sampled routinely performed in the ED with a level ≥2 µg/L. This PCT threshold was chosen upon previous studies observations, where ED patients with PCT ≥2 µg/L were suggested as having a sepsis and high risk of development of organ dysfunction [4, 8]. For each patient, ED electronic file and recorded admission data (including initial vital variables, routine biological data, and admitting diagnosis in ED) were collected, as well as outcome (discharge, admission to a medical ward or ICU, in-hospital mortality) and final diagnosis, i.e., each patient had a confirmation of sepsis suspected in the ED (based on all medical charts and available microbiological data). The presence of systemic inflammatory response syndrome (SIRS), sepsis, or severe sepsis/septic shock criteria [9] was also recorded, either at ED admission or during follow-up. For this study, hyperlactatemia (>2 mmol/L) was used as a severe sepsis criterion (although not specific) [10]. However, patients with other causes of high lactate levels were not excluded.

Patients were further categorized according to two different outcomes: death at day 30, or a combined outcome of death and/or admission to ICU during the follow-up of day 30.

### PCT measurement

In Tenon hospital, PCT concentrations were analyzed using a sandwich immunoassay based on time-resolved amplified cryptate emission (TRACE) measurement (Kryptor analyzer; B.R.A.H.M.S. Thermo Fischer, Germany). In our laboratory, coefficients of variation (CV) for PCT were found to be <10 % at 0.28 and 10.8 µg/L. In Rouen hospital, procalcitonin concentrations were analyzed using an electrochemiluminescent immunoassay (ELECSYS BRAHMS procalcitonin, Hennigsdorf, Germany), performed on a Cobas e601 analyzer (Roche Diagnostics, Meylan, France). In our laboratories, coefficients of variation for procalcitonin were <4 % at 2 concentrations during the study period. Both methods are correlated [11]. In our laboratories, correlation coefficient was R^2^ = 0.964 (slope: 1.04, intercept: −0.73), and we observed 96.4 % of concordance between the two methods (data not shown). Median bias observed between the two methods was 11 %, which is acceptable [12]. The upper reference limit (URL) announced by the manufacturer was 0.046 µg/L.

### Statistical analysis

Results were expressed as medians [interquartile range, IQR] for continuous variables and numbers (percentage) for discrete variables. Data were compared using the Kruskal–Wallis test for continuous variables, and the Chi-square for differences in frequencies.

Receiver operating characteristic (ROC) analysis was performed in order to determine the best thresholds for PCT (or the combination of PCT + lactate after log transformation) which would be predictive of the outcome. Because of the possible impact of sample size on threshold value, a bootstrap analysis (1000 random samples with replacement) was performed to obtain a calculation of the optimal threshold of PCT and its 95 % confidence interval [13]. We assessed the sensitivity and specificity, positive (PPV) and negative predictive value (NPV) (all with their 95 % confidence intervals [95 % CI], calculated with the Wilson’s score with correction of continuity) for thresholds.

As the ROC curve is recognized to be potentially insensitive, the net reclassification index (NRI) method was used, as described [13]. For tests with binary outcomes, NRI is the same as the gain in certainty of the first test minus the gain in certainty of the second test, or alternatively stated, the differences in the sum of the sensitivity and specificity:

$$ {\text{NRI}}_{{{\text{second}}\;{\text{test}}\;{\text{vs}}\;{\text{first}}\;{\text{test}}}} = \left( {{\text{Sensitivity}} + {\text{Specificity}}} \right)_{{{\text{second}}\;{\text{test}}}} - \left( {{\text{Sensitivity}} + {\text{Specificity}}} \right)_{{{\text{first}}\;{\text{test}}}} $$

Since NRI is a powerful statistical tool, significant results might only have a poor clinical impact. In order to illustrate the improvement given by lactate in association with PCT, we provide a reclassification table that enables us to quantify the benefit of the association in terms of number of patients correctly reclassified. Furthermore, the reclassification table offers a practical representation of both the relationship between false positive and false negative, and the magnitude of the gain of predictability in quantitative terms (number of patients).

We further evaluated PCT and lactate combination using the best linear combination (BLC) method [14]. Briefly, this method relies on the creation of a formula in which PCT and lactate are moderated by their coefficient of covariance in the studied population. The obtained combination gives a score for each patient that can be studied as a biomarker by itself and submitted to ROC analysis and logistic regression.

A forward logistic regression was performed to assess variables associated with outcome. For this analysis, PCT levels were evaluated as categorical variables based on the optimum cutoff point previously determined by ROC curve. We adopted a conservative approach and only included significant preoperative variables in the univariate analysis (p value of entry <0.10). The discriminate power of the logistic regression was evaluated by the c-statistic (concordance index) and the goodness of fit of the model by the Hosmer–Lemeshow test.

The results were analyzed using Med Calc 3.4.2.0 for Windows (MedCalc Software, Mariakerke, Belgium). All tests were two-tailed. A *p* value <0.05 was considered to be statistically significant.

## Results

Main characteristics of the studied population (*n* = 188) are presented in Table 1. As expected, main sources of sepsis were pulmonary and urinary infections. Forty-three percent of patients had a severe sepsis/septic shock, and 28 % were transferred in ICU. Briefly, patients who deceased within 30 days (20 %; *n* = 37) were older, presented an initial higher cardiac rate and respiratory rate, had more comorbidity, had more frequently severe sepsis or septic shock, and presented higher lactate levels than survivals. Of note, there was no difference in age values between recruitment sites (*p* = 0.337). Ninety-one percent of patients were admitted to the hospital.

**Table 1:** Characteristics of the studied population

	All patients	Deceased at 30 days	Survival	p*
*n*	188	37	151	
Patients of Rouen [*n* (%)]	97 (52)	21 (57)	76 (50)	0.605
Patients of Tenon [*n* (%)]	91 (48)	16 (43)	75 (50)	vs Tenon
Age (years)	63 (51–80)	78 (63–83)	64 (46–78)	0.001
Men [*n* (%)]	101 (54)	22 (59)	79 (52)	0.551
Temperature (°C)	38.1 (37.1–39.0)	38.2 (37.1–39.8)	38.1 (37.0–38.8)	0.321
Systolic blood pressure (mmHg)	113 (96–137)	113 (93–136)	116 (97–137)	0.524
Diastolic blood pressure (mmHg)	70 (57–80)	65 (52–81)	72 (59–80)	0.260
Cardiac rate, in bpm	100 (87–118)	110 (95–129)	99 (85–116)	0.051
Respiratory rate (RR) (*n* = 74)	26 (20–34)	32 (29–40)	25 (20–32)	0.009
SpO_2_ in %	95.0 (92.5–98.0)	95.0 (91.0–97.5)	95.0 (93.0–98.0)	0.636
Personal history of:				
Cardiovascular disease [*n* (%)]	94 (50)	24 (65)	70 (46)	0.052
Respiratory disease [*n* (%)]	43 (23)	13 (35)	30 (20)	0.063
Other (chronic) [*n* (%)]	51 (27)	5 (14)	46 (30)	0.099
Immunosuppressors, corticoids, or chemotherapy [*n* (%)]	29 (15)	5 (14)	24 (16)	0.677
Bacterial infection [*n* (%)]	139 (74)	27 (73)	112 (74)	0.159
Pulmonary infection [*n* (%)]	59 (31)	7 (19)	52 (34)	0.085
Urinary infection [*n* (%)]	30 (16)	5 (14)	25 (17)	0.914
Abdominal infection [*n* (%)]	21 (11)	7 (19)	14 (9)	0.169
Skin/tissue infection [*n* (%)]	8 (4)	3 (8)	5 (3)	0.361
Meningitis [*n* (%)]	2 (1)	1 (3)	1 (1)	0.828
Several sites^a^	12 (6)	1 (3)	11 (7)	0.526
Other site^b^	7 (4)	3 (8)	4 (3)	0.264
Viral infection [*n* (%)]	3 (2)	1 (3)	2 (1)	0.835
Other infection [*n* (%)]^c^	46 (24)	9 (24)	37 (25)	0.669
Severe sepsis/septic shock [*n* (%)]	81 (43)	22 (59)	59 (39)	0.040
Median lactate (mmol/L) (*n* = 103)	2.2 (1.5–3.2)	3.1 (2.2–4.4)	2.1 (1.5–2.8)	0.003
Median white blood cells (G/L)	12.1 (7.9–18.4)	12.1 (9.0–17.4)	12.0 (7.5–18.7)	0.966
Hospital admission [*n* (%)]	172 (91)	33 (89)	139 (92)	0.818
Admission in ICU [*n* (%)]	52 (28)	14 (38)	38 (25)	0.181
Length of hospitalization (days)	9 (5–14)	5 (2–10)	10 (6–16)	<0.0001

Results are in mean ± SD, median (25th–75th percentile), or number (percentage)

* Between patients deceased and survival

^a^Mainly pulmonary and urinary concomitant bacterial infection

^b^Bacterial infection (ENT, blood stream infection from unknown origin)

^c^Non-bacterial infection (i.e., parasitic, fungic, or mycobacterium infection)

Patients with severe sepsis/septic shock had higher cardiac rate (110 [94–125] vs. 96 [84–110], *p* = 0.001) and respiratory rate (30 [24–38] vs. 20 [18–26], *p* = 0.003), higher lactate (2.4 [1.7–3.6] vs. 1.9 [1.5–2.8] mmol/L, *p* = 0.053), and lower diastolic blood pressure (62 [51–75] vs. 76 [64–84], *p* < 0.0001), lower systolic blood pressure (99 [88–119] vs. 129 [107–148], *p* < 0.0001), and higher SpO_2_ (96 [94–98] vs. 94 [91–98] %, *p* = 0.041), than those without severe sepsis/septic shock. Furthermore, patients admitted to ICU were younger (60 [49–81] vs. 69 [52–83] years, *p* = 0.011), had lower diastolic blood pressure (62 [50–76] vs. 73 [61–81] mm Hg, *p* = 0.005), lower systolic blood pressure (100 [90–126] vs. 120 [100–139] mm Hg, *p* = 0.005), higher respiratory rate (32 [27–39] vs. 24 [20–32], *p* = 0.010), higher lactate (2.5 [2.0–3.4] vs. 2.1 [1.5–2.9] mmol/L, *p* = 0.039), and presented more frequently severe sepsis/septic shock (68 vs. 38 %, *p* = 0.001), than patients not admitted to ICU.

**Fig. 1:**
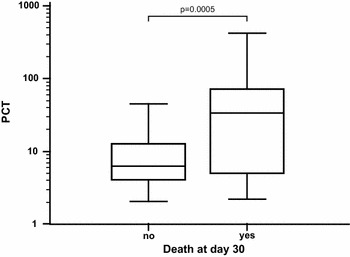
PCT values according to 30-day mortality

The median PCT value was higher in the deceased group (median [interquartile range, IQR]) (34.0 µg/L [5.0–71.9]) in comparison with the survivals (6.4 [4.1–13.1] µg/L, *p* = 0.0005) (Fig. 1). However, PCT values were not significantly higher in patients admitted to ICU in comparison with the others (9.6 [4.1–18.2] vs. 6.1 [3.9–14.1] µg/L, *p* = 0.145); same observation was done when comparing PCT values between patients with severe sepsis/septic shock versus others (8.6 [4.9–23.7] vs. 6.2 [3.6–12.3] µg/L, *p* = 0.064). Of note, there was no difference in PCT values between recruitment sites (*p* = 0.674).

### ROC analysis

**Fig. 2:**
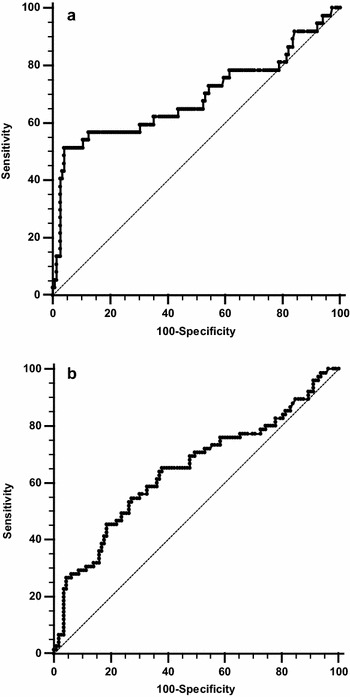
AUC of PCT to predict 30-day mortality (**a**) or to predict death and/or admission in ICU (**b**)

The ROC curve analysis indicated a moderate accuracy, with an AUC at 0.686 [95 % CI 0.613–0.752] (*p* = 0.002) for PCT to predict 30-day mortality (Fig. 2a). Defined by the ROC curve, the optimal threshold value was 32.5 [95 % CI 21.8–43.3] µg/L and was associated with a 51 % [34–67] sensitivity, a 96 % [90–98] specificity, a 73 % [52–88] PPV, and a 89 % [83–93] NPV. Only 26 patients (14 %) had PCT values above this threshold. The proportion of patients with PCT > 32.5 µg/L in the deceased group was significantly higher (51 %, *n* = 19) than in the survival group (5 %, *n* = 7) (*p* < 0.0001). However, the proportion of patients with PCT > 32.5 µg/L in the severe sepsis/septic shock (19 %, *n* = 16) was not significantly different from the proportion of patients with PCT > 32.5 µg/L in patients without severe sepsis/septic shock (10 %, *n* = 12) (*p* = 0.123).

The ROC curve analysis indicated a moderate accuracy for PCT to predict death and/or admission in ICU, with an AUC at 0.644 [0.571–0.712] (*p* = 0.007), with an optimal threshold value at 9.9 µg/L (Fig. 2b). This threshold was associated with a 54 % [42–65] sensitivity, a 73 % [63–90] specificity, a 70 % [61–78] PPV, and a 57 % [45–68] NPV.

**Fig. 3:**
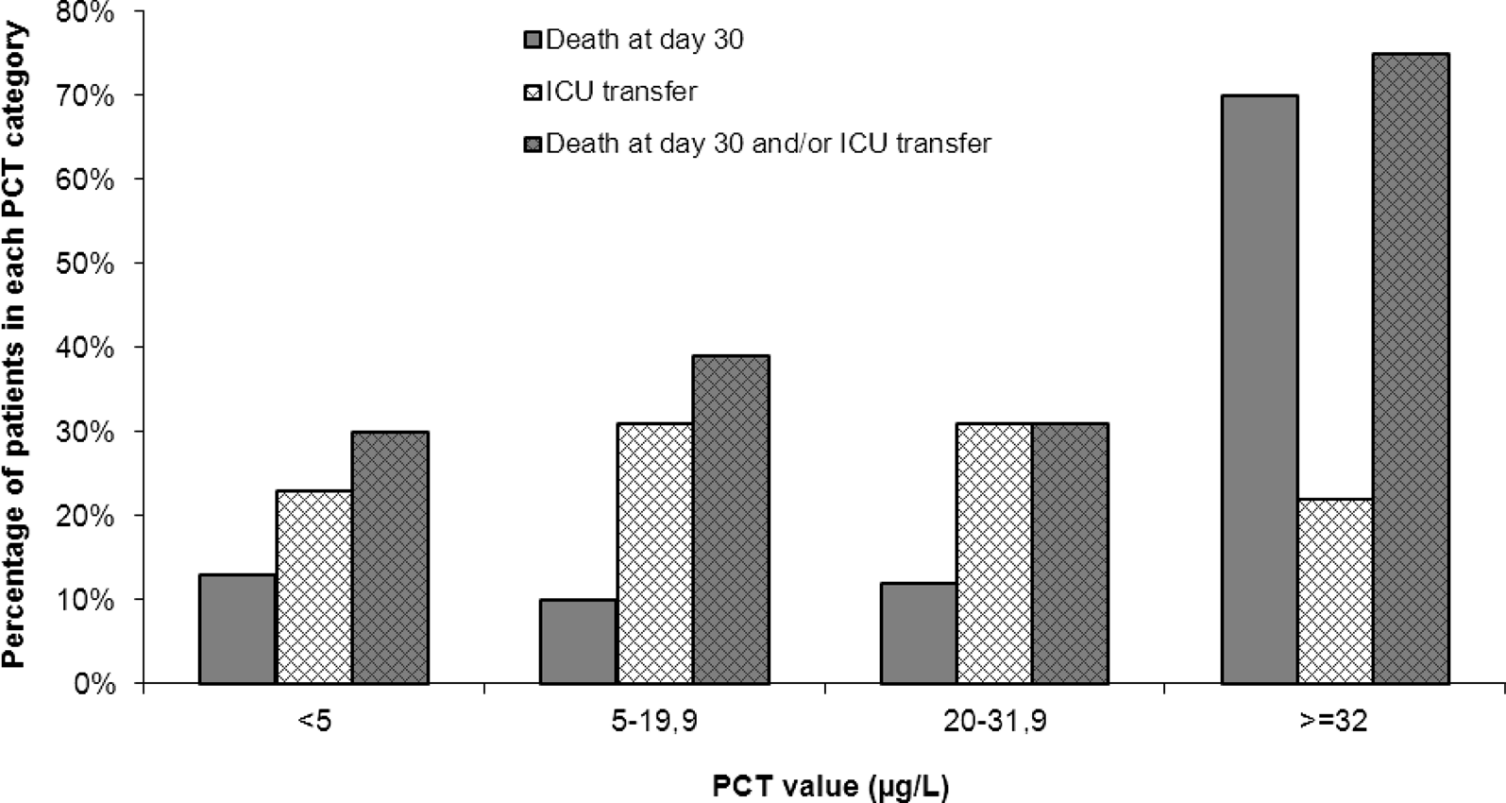
Outcomes (30-day mortality and/or combination of ICU transfer) according to levels of PCT

When patients were classified according to PCT categories (arbitrarily defined as PCT < 5 µg/L, PCT between 5 and 19.9 µg/L, PCT between 20 and 31.9 µg/L, and PCT ≥ 32 µg/L), we observed that mortality raised dramatically only in patients with PCT ≥ 32 µg/L, while ICU transfer was not affected by PCT value (Fig. 3).

### Analysis of subgroup in patients with available lactate measurement

When considering patients with available lactate measurement (*n* = 103), patients with PCT > 32.5 µg/L presented higher median lactate values (3.3 [1.9–4.3] mmol/L), in comparison with patients with lower PCT (2.1 [1.5–3.0] mmol/L, *p* = 0.0347).

When combining PCT and lactate log-transformed values, the ROC curve analysis indicated an AUC at 0.692 [95 % CI 0.594–0.780] for the prediction of 30-day mortality. This was significantly higher than PCT alone in this subgroup (*p* = 0.020). However, sensitivity (50 %) and specificity (96 %) were similar to those obtained with PCT alone. Alternatively, using the BLC method, we obtained the following combination: PCT (in µg/L) + 0.025 × lactate (in mmol/L). This combination gives a score for each patient and was tested in ROC analysis. Unfortunately, it failed to maximize ROC curve (AUC = 0.633 [0.533–0.726] vs. PCT alone, *p* = 0.061).

**Fig. 4:**
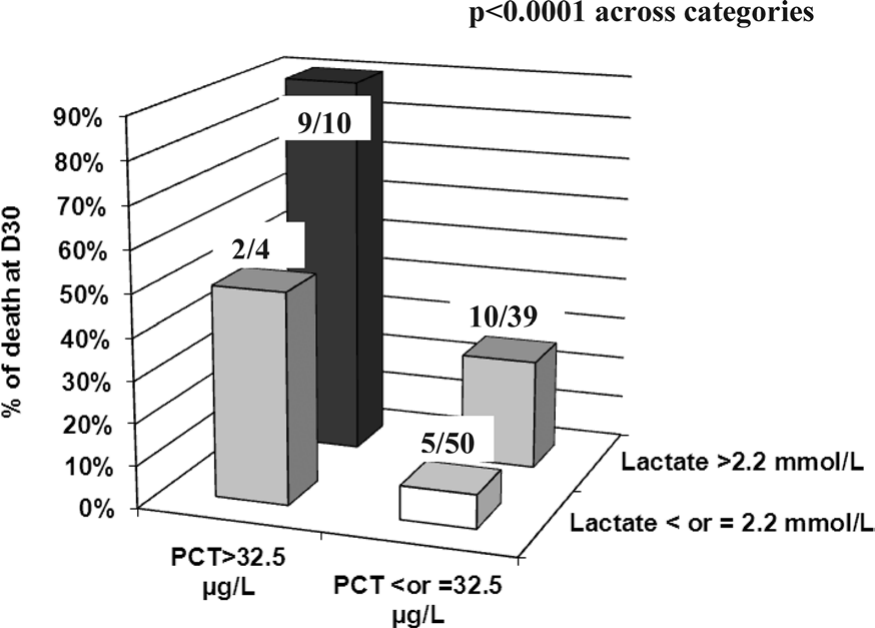
Outcome according to PCT and lactate thresholds. PCT and lactate thresholds were given by ROC analysis. *Ratio* indicates number of deceased patient at day 30 over number of patients in each category

Patients with both PCT > 32.5 µg/L and lactate >2.2 mmol/L (i.e., above ROC thresholds) presented the worse outcome if considering death at day 30 (*p* < 0.0001) (Fig. 4). Using these thresholds, a table of contingency was built (Table 2), and NRI was calculated. The NRI was 0.8 % (*p* = 0.980), indicating that patients were not significantly better identified as at risk of death using lactate on top of PCT alone.

**Table 2:**
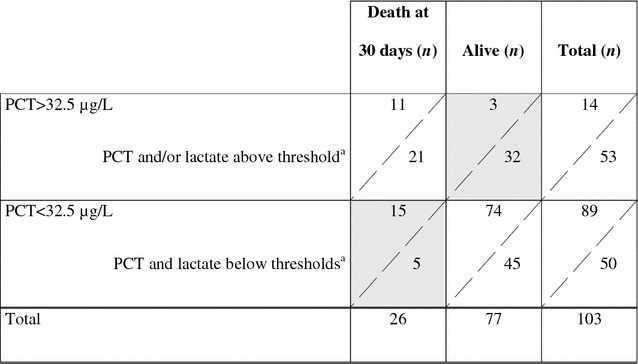
Net reclassification table of patients according to PCT and lactate values

NB. This is the practical representation of both the relationship between false positive and false negative (gray zones), and the magnitude of the gain of predictability in quantitative terms (number of patients): here, the strategy PCT + lactates provokes a decrease in false negative (from 15 to 5) but a concomitant increase in false positives (from 3 to 32). The consequence is that sensitivity is improved (from 11/26 = 42.3 % to 21/26 = 80.8 %), but consequently specificity is dramatically decreased (from 74/77 = 96.1 % to 45/77 = 58.4 %)

Thus, the NRI calculation is: NRI = (80.8 + 58.4) − (42.3 + 96.1) = 0.8 %

^a^32.5 µg/L for PCT, 2.2 mmol/L for lactate

When considering the prediction of death and/or admission in ICU, the combination of PCT and lactate indicated an AUC at 0.677 [95 % CI 0.578–0.765], which was significantly higher than PCT alone in this subgroup (*p* = 0.012). However, sensitivity (50 %) and specificity (73 %) were similar to those obtained with PCT alone.

### Multivariate analysis

Multivariate analysis was performed on the entire study group and is presented in Table 3. Three variables remained independently and significantly predictive of death at day 30: previous history of cardiovascular disease, the presence of severe sepsis/septic shock, and a PCT > 32.5 µg/L, which remained the strongest predictor of death. If considering prediction of death and/or admission to ICU, a PCT value >32.5 µg/L also remained the strongest independent and significant predictor, with an OR value at 6.4 [2.3–17.9] (*p* = 0.0004), with the presence of severe sepsis/septic shock (OR 4.4 [2.2–8.9]).

**Table 3:** Univariate and multivariate analysis for independent prediction of death at 30 days

	Univariate analysis	*p*	Multivariate analysis	*p*
	OR [95 % CI]		OR [95 % CI]	
Age	1.04 [1.01–1.06]	0.0015	/	
Heart rate	1.02 [1.00–1.03]	0.047	/	
Personal history of:				
Cardiovascular diseases	2.5 [1.1–5.7]	0.024	3.1 [1.0–9.4]	0.0462
Respiratory disease	2.4 [1.05–5.2]	0.036	/	
“Other” chronic diseases	0.4 [0.1–1.0]	0.052	/	
Severe sepsis/septic shock	2.3 [1.1–4.8]	0.027	4.0 [1.3–12.3]	0.0130
PCT > 32.5 μg/L	21.7 [8.0–58.8]	<0.0001	36.0 [10.0–128.4]	<0.0001

As data were partially collected for lactate and respiratory rate, these parameters could not be included in the analysis

If considering prediction of death and/or admission to ICU, the PCT + lactate score remained the only independent and significant predictor of death, with an OR value at 6.0 [95 % CI 1.3–28.5] (*p* = 0.024).

## Discussion

We aimed to evaluate in the emergency setting the prognostic value of PCT in septic patients. Our results demonstrated that PCT is a potential tool by itself for early identification of ED septic patients with poor outcome (death at day 30). However, its prognostic accuracy is not useful enough, to be used in daily practice.

A recent meta-analysis indicated that elevated PCT concentrations are associated with all-cause mortality in septic patients. However, studies are still lacking to define the optimal cutoff point, especially in ED patients [1]. This previous meta-analysis pointed out the heterogeneity of the results observed in the studies. In the specific field of ED patients, there are few studies that report association of PCT level with mortality [15–17]. Authors present heterogeneity in methodologies (fully automated or semiquantitative assays), in reported cutoffs (from 0.9 to 10 µg/L), and in testing time (day 0, 1 or 5). In addition to these data, the study of Freund et al. [3] indicated, with another fully automated method for PCT (Kryptor Brahms), a cutoff at 0.8 µg/L for identification of severe outcome in the ED. Thus, relationship between PCT and prognosis required further study.

To our knowledge, our study is the first to focus on patients with high-PCT values (above 2 µg/L) in emergency septic patients. Our results suggest a moderate relation between PCT level and outcome, in the specific population of septic patients with high PCT. As expected, we find a higher cutoff than previous studies performed in ED [3, 15–17], but we also had a better specificity. In terms of patients’ recruitment, our results should be compared to those of Hur et al. [7] who investigated the diagnostic and prognostic utility of PCT in critically ill patients with suspected sepsis in ED and ICU, for whom sepsis was diagnosed clinically or on PCT concentrations. Thus, our results are in accordance with those of Hur et al. [7], as we found similar PCT values in non-survivors (mean PCT at admission around 32 µg/L).

When considering our ROC curve analysis for death prediction, our results are similar to those of Zhao et al. [18] and to those of Wang et al. [19], but with a higher cutoff. In our population, we observe similar performances with the solely PCT, in comparison with that of Zhao when using MEDS score +PCT [18], or to that of Wang using PCT alone [19]. Wang et al. [19] found a 28-day mortality cutoff at 4.3 µg/L, which is lower than ours, but they included both septic patients and SIRS patients without infection. MEDS score has been suggested as a score system with high ability to predict the 28-day outcome of ED patients with systemic inflammatory response syndrome (SIRS), sepsis, or severe sepsis [20–22]. However, some studies indicated that MEDS score is not suitable for patients with severe sepsis or septic shock [23, 24].

We found that PCT is a moderate predictor of 30-day mortality but also for the prediction of death and/or admission in ICU in confirmed septic ED patients. Our results are in accordance with most of the previous studies [4, 7, 15–18]. However, some studies also failed to demonstrate any prognostic value of PCT [19]. In our septic population, the PCT value for predicting 30-day mortality is higher (32.5 vs 4.3 µg/L), with a better specificity (96 vs 84 %) than previously observed [19]. However, this strategy allows to target a small number of patients in our population (*n* = 19 deceased patients with a PCT above 32.5 µg/L, corresponding to 51 % of the deceased patients but only 10 % of the whole study population), and thus the interest of its use in routine might be limited.

Furthermore, when considering the ROC curve analysis for the prediction of death and/or admission in ICU, prognostic performances were lower, and we did not find any significant difference in PCT values between patients admitted to ICU versus the others. This latest observation was somehow expected as many other factors (such as comorbidities including dementia, functional status, and bed availability…) are used in the decision to admit a patient in ICU. The weight of biological (as PCT) or physiological data is probably relatively low compared to other variables in the decision of ICU transfer, as reflected by our Fig. 3.

In our subgroup analysis, we did not observe any additional performance when combining lactate to PCT, for the prediction of 30-day mortality or for the prediction of death and/or admission in ICU. This is not in accordance with results obtained by Freund et al. [3], who—in a larger sample of ED patients—considered a combined outcome (death and/or ICU admission and/or terminal patients with therapy limitations). However, our patients with both PCT > 32.5 µg/L and lactate >2.2 mmol/L presented the worst outcome, which suggests the potential complementary role of both biomarkers in identifying very high-risk patients. Unfortunately, using NRI and BLC methods, we failed in finding strong additional information in the association PCT + lactate. This might be because of the small subgroup number of patients and should be specifically investigated in a larger cohort.

### Limitations

Some limitations merit consideration in this study. Firstly, this is a bicentric retrospective, non-blind study, including a somehow limited number of patients. Secondly, we did not use severity score systems (MEDS, SOFA, etc.) to compare with, or in addition to PCT. Thirdly, the dynamic changes in biomarkers were not evaluated. Fourthly, we do not have data on previous antibiotherapy or antibiotics given in the ER or after. Thus, we do not have information on their adequacy. Fifthly, lactate was not measured systematically to all patients. Therefore, this variable could not be fully investigated and included in our multivariate analysis. Finally, two different methods were used to assay PCT. However, the two methods are known to be highly correlated [11, 12], and a minimal bias was observed in our population.

## Conclusion

Elevated value of PCT at admission has moderate accuracy to identify poor outcome in ED septic patients. We suggest that the measurement of PCT in ED septic patients should not be routinely performed to assess prognostic information, before other evaluation of its added value in further studies.
